# Coupling of Glucose Deprivation with Impaired Histone H2B Monoubiquitination in Tumors

**DOI:** 10.1371/journal.pone.0036775

**Published:** 2012-05-16

**Authors:** Yasuyo Urasaki, Linda Heath, C. Wilson Xu

**Affiliations:** 1 Nevada Cancer Institute, Las Vegas, Nevada, United States of America; 2 The Biological Research Institute, Pasadena, California, United States of America; Tulane University Health Sciences Center, United States of America

## Abstract

Metabolic reprogramming is associated with tumorigenesis. However, glucose metabolism in tumors is poorly understood. Here, we report that glucose levels are significantly lower in bulk tumor specimens than those in normal tissues of the same tissue origins. We show that mono-ubiquitinated histone H2B (uH2B) is a semi-quantitative histone marker for glucose. We further show that loss of uH2B occurs specifically in cancer cells from a wide array of tumor specimens of breast, colon, lung and additional 23 anatomic sites. In contrast, uH2B levels remain high in stromal tissues or non-cancerous cells in the tumor specimens. Taken together, our data suggest that glucose deficiency and loss of uH2B are novel properties of cancer cells *in vivo*, which may represent important regulatory mechanisms of tumorigenesis.

## Introduction

Cancer cells exhibit aberrant glucose metabolism characterized by aerobic glycolysis, a phenomenon also known as the Warburg effect [1], [2]. This metabolic reprogramming is thought to play an important role in supplying proliferating tumors with necessary building blocks for biomass production [3]. Compelling evidence also indicates that oncogenes and tumor suppressors play opposing roles in regulating glucose metabolism [3]. Despite the importance of glucose metabolism in tumors, it is not known whether high glucose levels are required for cancer cells to maintain their proliferative advantage *in vivo*.

## Results and Discussion

**Figure 1:**
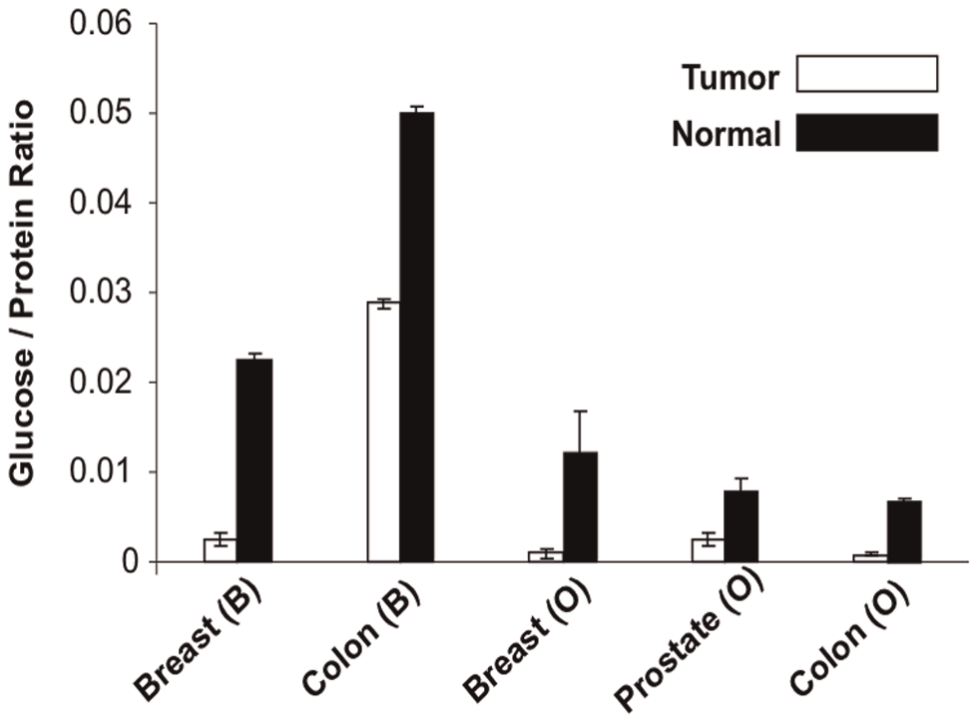
Glucose levels in tumors are lower than those of normal tissues of the same tissue sites. Five pairs of matched tumor and normal tissue specimens from Biochain (B) and Origene (O) were analyzed for glucose and protein content. The amount of glucose was normalized with the total protein concentration. Each sample was assayed in quadruplicate.

To determine glucose levels in human tumors and normal tissues, we first assayed the glucose contents in matched clinical tumor specimens and normal tissues of the same tissue origins. Because of the inherent variability of clinical tissue specimens [4], we normalized the glucose levels with the total protein from the matched specimens, an approach that has been adopted by others [5]. Although it is difficult to estimate the total protein levels in cancer and normal cells as a result of the tumor heterogeneity, the total protein level in tumor interstitial fluid is comparable to that of normal subcutaneous fluid in xenograft models [6], suggesting that the total protein levels might be operationally useful for normalizing the glucose amounts in inherently-variant clinical tumor and matched normal tissue specimens. As shown in Fig. 1, the relative amounts of glucose from frozen and unfixed human breast, prostate and colon tumor specimens were much lower than those of normal cells of the same tissue sites, indicating that glucose may be deprived in the bulk tumor specimens. These results are consistent with the observation that lower amounts of glucose are detected in tumor veins than in tumor arteries in rats [2]. These results are also in agreement with the fact that lower amounts of glucose are found in tumor interstitial fluid than in normal subcutaneous interstitial fluid in xenograft models [6]. Moreover, these results are consistent with the finding that glucose levels, detected by low-resolution bioluminescence assays, are drastically increased in bulk tumor specimens that have been treated with chemotherapy or radiation in comparison to untreated tumors in a xenograft model for lung cancer [7].

**Figure 2:**
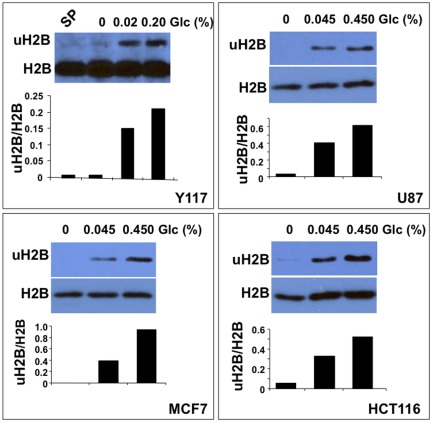
uH2B is a semi-quantitative histone marker for glucose. Stationary phase (SP) yeast (Y117) was incubated with different amounts of glucose for 1 hr. Brain (U87), Breast (MCF7) and colon (HCT116) cancer cell lines were grown in complete media (10% FBS/DMEM) until 40–60% confluency and subsequently incubated with DMEM containing 10% dialyzed FBS with indicated amounts of glucose for 24 hrs. The glucose (Glc) concentrations used in the assays covered the physiological serum glucose levels (0.07–0.12%). uH2B levels in these cells corresponded to those of glucose semi-quantitatively. Half of the glucose-treated tumor cells were also formalin-fixed and paraffin-embedded for immunohistochemical staining of uH2B and H2B (Fig. S1). uH2B levels, detected by immunohistochemistry, were also proportional to those of glucose, further suggesting that uH2B may be used as a chromatin marker for glucose.

Tumors are typically heterogeneous organs with a microenvironment of various non-malignant cell types both within the tumor area and in their stromal environment [8]. Therefore, we reasoned that a glucose marker would be required to identify the cellular source of glucose deprivation in the bulk tumor specimens. We have previously demonstrated that glucose is the sole nutrient inducer of mono-ubiquitination of histone H2B (uH2B) at K123 in yeast, and at its orthologous site K120 in human cells [9], [10], [11], indicating that uH2B is an evolutionarily conserved chromatin marker for glucose. To test whether uH2B could be used as a semi-quantitative marker for glucose, we grew U87 (glioblastoma), MCF7 (breast cancer) and HCT116 (colon cancer) in various amounts of glucose spanning serum normal glucose levels. We then analyzed the levels of uH2B in these cells with an antibody specific to ubiquitinated histone H2B at K120 [12]. As shown in Fig. 2, exposure to increasing levels of glucose resulted in a corresponding increase in levels of uH2B in tumor cells. In contrast, H2B levels remained unchanged in all samples. These data suggest that uH2B can be used as a semi-quantitative histone marker for glucose in tumor cells.

We have reported that uH2B is not detectable in stationary phase yeast by Western blotting analysis [9]. Using a yeast strain (Y117) with FLAG-H2B as the sole source of H2B, we incubated stationary phase Y117 in various amounts of glucose for 1 hr [9]. As shown in Fig. 2, the uH2B levels also correlated with those of glucose, whereas H2B remained unchanged in all samples. Taken together, these data further suggest that uH2B is an evolutionarily conserved semi-quantitative marker for glucose in yeast and tumor cells.

We noted that uH2B levels as a function of glucose concentration were not linear in the range of glucose concentrations that we tested. Further analysis would clarify whether some of the glucose concentrations were at or above the saturation point for some of the cells or whether it is due to the nonlinearity of Western blotting analysis, which was based on chemiluminescence/X-ray film imaging. Nevertheless, our results indicate that uH2B levels correlate semi-quantitatively with those of glucose in yeast and tumor cells.

To determine whether the uH2B levels as a function of relative amounts of glucose could be detected by immunohistochemistry, we formalin-fixed and paraffin-embedded the same batches of glucose-treated tumor cells used for the Western blotting analyses in Fig. 2. After hybridizing the cellblock sections with antibodies specific to either uH2B or H2B, and horseradish peroxidase-conjugated secondary antibody, we then counterstained the cells with Hematoxylin. At least 1000 cells were examined for each sample. As shown in Fig. S1, uH2B levels detected by immunohistochemistry correlated with the amounts of glucose that the cells were exposed to. In contrast, H2B levels remained unchanged in all samples. Taken together, these data further demonstrate that glucose-induced uH2B may be used as a semi-quantitative chromatin marker for examining relative amounts of glucose in tumor specimens from cancer patients.

**Figure 3:**
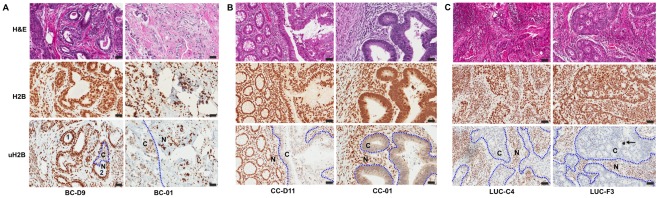
Glucose-induced uH2B is significantly impaired in cancer cells compared to their adjacent stromal tissues. Human breast, colon and lung tumor specimens from surgery were immunohistochemically stained for uH2B and H2B and subsequently counter-stained with Hematoxylin. **A**. uH2B levels are inhibited in breast cancer cells in 36 out of 37 cases. Two representative cases are shown. Intense staining of uH2B was observed in normal myoepithelial and luminal epithelial cells of Duct 1 of a tumor specimen from breast cancer patient BC-D9. Although it was in the tumor specimen, Duct 1 had no cancer cells. uH2B was significantly reduced in luminal epithelial cancer cells, which was encircled with a dashed line in Duct 2. In contrast, uH2B remained high in normal luminal epithelial cells in Duct 2. Other cancer cells in BC-D9 tumor specimen were not separated with dashed lines for the purpose of clarity. Breast cancer cells from patient BC-01 also showed low uH2B staining, whereas adjacent normal cells maintained high uH2B staining. **B**. uH2B levels are drastically reduced in colon cancer cells in 35 out of 36 cases. Two representative cases are shown. **C**. uH2B levels are significantly inhibited in lung cancer cells in 35 out of 36 cases. Two representative cases are shown. The black arrow shows a piece of cigarette tar. N denotes normal cells or stromal tissues. C denotes cancer cells. Dashed lines demarcate cancer cells from their adjacent normal or stromal cells. Scale bar  = 50 µm.

To identify a cellular source of glucose deprivation observed in the bulk tumor specimens (Fig. 1), we examined glucose-induced uH2B levels from patient biopsies or surgery specimens. As shown in Fig. 3a, breast cancer cells showed significantly less uH2B staining than their adjacent stromal cells. uH2B levels also exhibited a clear demarcation between cancer cells and their adjacent normal cells. For instance, uH2B staining was intense in both myoepithelial and luminal epithelial cells in normal breast duct (Duct 1, BC-D9 breast cancer specimen, Fig. 3a). However, uH2B levels were significantly reduced in luminal epithelial cancer cells that had undergone transformation while remained unchanged in normal luminal epithelial cells in Duct 2. In contrast, H2B levels were the same in both normal and cancer cells. In another breast cancer case, uH2B levels were also lower in cancer cells while uH2B levels remained high or unchanged in adjacent normal tissue (BC-01, Fig. 3a). Similarly, uH2B levels were also lower in cancer cells compared to their adjacent non-cancer cells in 33 cases of additional 34 breast cancer specimens of different histopathological types, grades and stages.

To determine whether impairment of glucose-induced uH2B occurs in other types of cancer cells, we analyzed tumor specimens from colon and lung cancer patients. As shown in Fig. 3b and 3c, impairment of uH2B was also evident in colon and lung cancer cells. Specifically, of 36 colon tumor specimens of various types, grades and stages, 31 cases showed lower uH2B levels in cancer cells compared to their stromal cells. Weak uH2B levels were also observed in 4 tumor specimens, in which no stromal cells were present. Moderate uH2B levels in one case were detected in both cancer and their stromal cells.

Of 36 lung cancer specimens of various types, grades and stages, 35 cases showed lower uH2B levels in cancer cells compared to their adjacent stromal cells. One case showed low levels of uH2B in all cells of the specimen, in which no stromal cells were present. Therefore, impairment of uH2B was detected in virtual all breast, colon and lung cancer cells compared to their stromal cells in 106 out of 109 cases. Furthermore, lower uH2B levels were also observed in cancer cells of additional 23 anatomic sites compared to their stromal tissues (n = 1−3 per anatomic site, data not shown). Taken together, these results indicate that impairment of glucose-induced uH2B is characteristic of cancer cells *in vivo.*

As discussed earlier, glucose is the sole nutrient inducer of uH2B in yeast and mammalian cells [9], [11]. uH2B levels correlated with the amounts of glucose in cultured cells by both Western blotting and immunohistochemical analyses (Fig. 2 and Fig. S1). Therefore, lower levels of uH2B in cancer cells of the tumor specimens may thus represent glucose deprivation in cancer cells *in vivo*. This is consistent with the observation that relative glucose levels were lower in bulk tumor specimens than those of normal cells of the same tissue origins (Fig. 1). As discussed above, uH2B levels exhibit a clear demarcation between cancer cells and their adjacent normal cells (Fig. 3). In addition, loss of uH2B occurs, to a similar extent, in all cancer cells within a cancer cell nest (Fig. 3). Taken together, these data thus suggest that glucose deficiency is characteristic of cancer cells *in vivo*.

Glucose deprivation in cancer cells may be a molecular basis for clinical detection of tumors by positron emission tomography (PET). PET depends on the fact that tumors exhibit higher uptake of ^18^F-deoxyglucose. Since ^18^F-deoxyglucose uptake inversely correlates with glucose concentrations in cultured cells [13], PET detection of ^18^F-deoxyglucose uptake in tumors may reflect glucose deprivation in cancer cells. Because glucose deficiency can select cells with oncogenic mutations *in vitro* [14], glucose deprivation of cancer cells *in vivo*, as demonstrated in this study, may offer a proliferative advantage of cancer cells in patients.

We have previously reported that glucose-induced uH2B regulates expression of metabolic genes in yeast [9]. Others have recently shown that uH2B is also required for DNA repair in yeast and mammalian cells [15], [16], [17]. Since uH2B was impaired in virtually all cancer cells from breast, colon, lung and additional 23 anatomic sites that we have tested, coupling of glucose deprivation with loss of uH2B may likely play an important role in regulating metabolic reprogramming and DNA damage response in tumorigenesis.

## Materials and Methods

### Cell Lines, Culture Media, Chemicals and Antibodies

U87 MG human glioblastoma (grade IV) cells and MCF7 (ATCC) were maintained in high-glucose Dulbecco’s modified Eagle’s medium (DMEM) (4.5 g/L glucose, 0.584 g/L glutamine and 110 mg/L pyruvate, (catalog # 11995, Gibco) supplemented with 10% fetal bovine serum (catalog # A15–351, PAA Laboratories) and 1% penicillin/streptomycin (P/S) at 37°C in a humidified atmosphere of 95% air and 5% CO_2_. LnCap and HCT116 (ATCC) were cultured in RPMI 1640 medium (catalog # 11875, Gibco) supplemented with 10% FBS and 1% P/S at 37°C in a humidified atmosphere of 95% air and 5% CO_2_. FBS (10 ml) was dialyzed against PBS (pH 7.4, 2×1 liter) at 4°C for 48 hrs. The dialyzed FBS (dFBS) was filtered through 0.22 µm filter unit (Millipore) and stored at 4°C until use.

Glucose minus DMEM (catalog # 11966, Gibco) contained 584 mg/L L-glutamine but no glucose. Glucose minus RPMI 1640 (catalog # 22400, Gibco) contained L-glutamine but no glucose. Mouse monoclonal antibody specific to ubiquitinated histone H2B at K120 was from Medimabs (catalog # MM-0029-P) [12]. Histone H2B antibody (ChIP grade) was from Abcam (catalog # ab1790). Beta-actin antibody was from Abcam (catalog # ab8224). Peroxidase-conjugated immuno pure goat anti-mouse IgG (H+L) (catalog # 31430) and peroxidase-conjugated immuno pure goat anti-rabbit IgG (H+L) (catalog # 31460) were purchased from Pierce.

### Glucose Analysis of Human Normal Tissue and Tumor Samples

Matched human tumor and normal tissue specimens of the same tissue origins (the samples were paired, unfixed and frozen) were from BioChain (Breast cancer, catalog # P8235090-PP; and colon cancer, catalog # P8235090-PP). Additional paired tumor and normal specimens (frozen/unfixed breast, prostate and colon tumor/normal tissues CP5656504; CP565671; CP5655651; CP565424; CP5655718; CP565608) were from Origene. Glucose was assayed as previously described [9]. Protein concentration was estimated with CB-X Protein Assay Kit (catalog # 786-12X, G Biosciences).

### Analysis of Glucose-regulated uH2B in Cultured Tumor Cells and Yeast

U87 (Glioblastoma), MCF7 (breast cancer), LnCap (prostate cancer), and HCT116 (colon cancer) were cultured in 60 mm dishes with high-glucose DMEM or RPMI 1640 supplemented with 10% FBS and 1% P/S until they were 40–60% confluent. After the media were removed, cells were rinsed twice with phosphate buffered saline (PBS) and subsequently incubated with glucose minus medium DMEM (catalog # 11966) or RPMI 1640 (catalog # 22400) supplemented with 10% dialyzed FBS and 1% P/S and 0%, 0.045% or 0.450% glucose for 24, 40, or 48 hrs. Although some cells became detached during the glucose-minus medium incubation, virtual all of the detached cells excluded Trypan Blue (data not shown), suggesting that they were alive.

To collect both attached and detached cells, we scraped the attached cells in glucose minus medium with cell scrapers (catalog # 353085, BD) and collected the cell suspension by centrifugation at 200 g for 2 min. The cell pellets were boiled in 4 X SDS-PAGE sample buffer at 100°C for 5 min. Protein concentration was estimated with CB-X Protein Assay Kit (catalog #786-12X, G Biosciences) and normalized by Western blotting. For yeast analysis, stationary phase (SP) yeast (Y117), which contained FLAG-tagged H2B as the sole source of H2B (submitted), was incubated with different amounts of glucose for 1 hr and harvested for Western blotting analysis as described [9]. The intensity of Western blotting signals was estimated with Image J.

To correlate Western analysis with immunohistochemical analysis, half of the harvested cells were formalin-fixed and paraffin-embedded. Specifically, cell suspension were centrifuged at 200 g for 2 min and washed once with PBS. Washed cells were collected by centrifugation and re-suspended by 1∶10 buffer diluted formalin at room temperature for 24 hrs, subsequently paraffin-embedded and immunohistochemically stained.

### Immunohistochemical Analysis of Clinical Tumor Specimens

Breast, colon and lung tumor arrays were from Pantomics (catalog # BRC962, COC962 and LUC962). Tumor arrays from 27 anatomic sites were from BioChain (catalog # Z7020082, lot # B412135). Mach 4 Universal HRP-Polymer Detection Kit (Biocare Medical, LLC) were used for immunohistochemical analyses with 200 x dilution of the antibody raised against a synthetic branch peptide of ubiquitinated histone H2B at K120 [12] (Medimabs, catalog # MM-0029-P) or histone H2B antibody (Abcam, catalog # ab1790). All images (40×) were captured and analyzed with an Aperio scanner (USC).

## Supporting Information

Fig. S1 **Tumor cells, from the same batches of the glucose-treated cells that were used in Western blotting analysis (** **Fig. 2** **), were formalin-fixed and paraffin-embedded for immunohistochemical staining of uH2B and H2B.** These cells were subsequently counterstained with Hematoxylin. At least 1000 cells were examined for each sample. uH2B levels correlated with the amounts of glucose (Glc) of the media for culturing glioblastoma cells (U87, **A**), breast cancer (MCF7, **B**) and colon cancer (HTC116, **C**) cells. Scale bar  = 50 µm.
(DOC)
