# Supplementary figures and images for: Coupling of Glucose Deprivation with Impaired Histone H2B Monoubiquitination in Tumors
Source: PLoS One. 2012 May 16;7(5):e36775. doi: 10.1371/journal.pone.0036775 (PMC3353945; doi:10.1371/journal.pone.0036775)

**Fig. S1A**

**
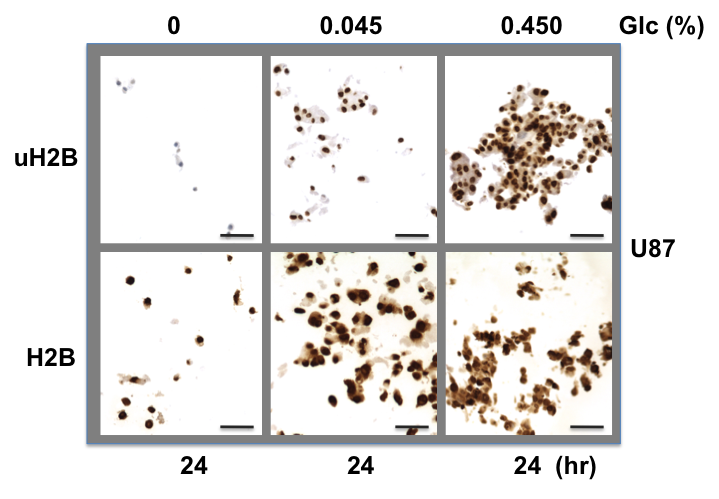
**

**Fig. S1B**

**
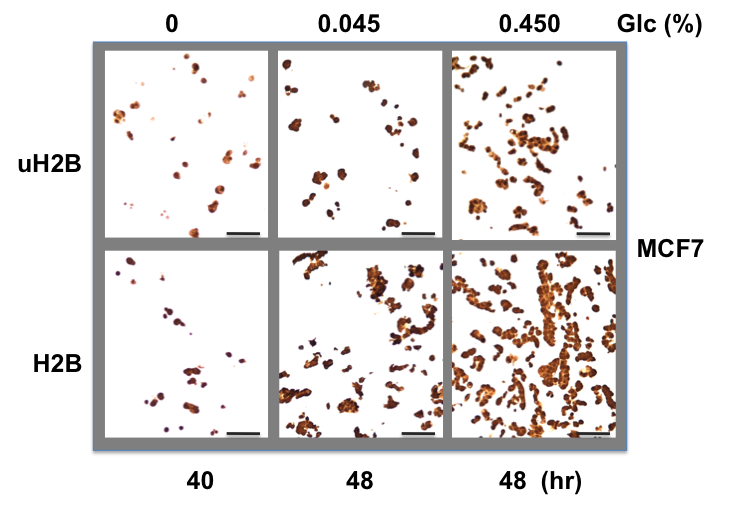
**

**Fig. S1C**


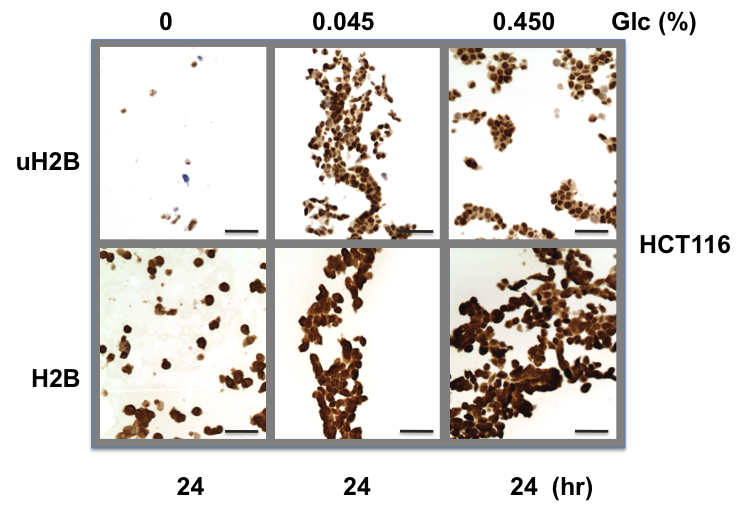

Supplement: Fig. S1 — Tumor cells, from the same batches of the glucose-treated cells that were used in Western blotting analysis ( Fig. 2 ), were formalin-fixed and paraffin-embedded for immunohistochemical staining of uH2B and H2B. These cells were subsequently counterstained with Hematoxylin. At least 1000 cells were examined for each sample. uH2B levels correlated with the amounts of glucose (Glc) of the media for culturing glioblastoma cells (U87, A), breast cancer (MCF7, B) and colon cancer (HTC116, C) cells. Scale bar = 50 µm. (DOC) [file pone.0036775.s001.doc]
